# Pyroptosis in NLRP3 inflammasome-related atherosclerosis

**DOI:** 10.15698/cst2022.10.272

**Published:** 2022-10-10

**Authors:** Xiang Zeng, Dongling Liu, Xia Huo, Yue Wu, Cuiqing Liu, Qinghua Sun

**Affiliations:** 1 School of Public Health, International Science and Technology Cooperation Base of Air Pollution and Health, Zhejiang Chinese Medical University, 548 Binwen Road, Hangzhou 310053, Zhejiang Province, China.; 2 School of Basic Medical Science, Zhejiang Chinese Medical University, 548 Binwen Road, Hangzhou 310053, Zhejiang Province, China.; 3 First Affiliated Hospital of Xinxiang Medical University, Weihui 453100, Henan Province, China.; 4 Laboratory of Environmental Medicine and Developmental Toxicology, Guangdong Key Laboratory of Environmental Pollution and Health, School of Environment, Jinan University, Guangzhou 511443, Guangdong Province, China.

**Keywords:** NLRP3 inflammasome, caspase-1, GSDMD, IL-1β, pyroptosis, atherosclerosis, cardiovascular diseases

## Abstract

Pyroptosis is a proinflammatory form of programmed cell death in response to inflammation. It involves in the pathogenesis and outcomes of atherosclerosis characterized by NLRP3 inflammasome assembly, membrane pore formation, cell swelling, pro-inflammatory mediator and cytokine release. There are known pyroptosis molecular pathways including the caspase-1 depended canonical signaling pathway and the caspase-4/5/11 determined non-canonical signaling pathway. It is essential to explore the connection among NLRP3 inflammasome, pyroptosis and atherosclerosis, which may shed light on the potential therapeutic strategies that target pyroptosis in atherosclerotic treatment.

## INTRODUCTION

Cardiovascular diseases (CVDs) are the leading causes of mortality and morbidity globally, which was estimated that 17.9 million individuals died due to CVDs in 2019, accounting for 32% of all global deaths [1]. Atherosclerosis is a cardiovascular progressive lesion occurred in coronary, cerebral, or peripheral vessels *etc.*, resulting in serious health threat, heavy social and economic burdens [2][3][4]. It is characterized by the thickening of intima and the formation of plaque at sites with endothelial cell injury and chaotic laminar flow, and has been regarded as a metabolic inflammatory disorder [5][6][7].

Nucleotide-binding oligomerization domain-like receptor (NLR) family pyrin domain containing 3 (NLRP3) inflammasome is an intracellular molecular platform consisting of NLRP3, ASC (apoptosis-associated speck-like protein containing a caspase recruitment domain) and pro-caspase-1, and may be activated by a diverse range of stimuli such as pathogens, irritants, apoptotic and pyroptotic cells [8][9][10]. Additionally, NLRP3 inflammasome plays a crucial role in inflammatory response to defend external stimuli. Potassium (K^+^) eflux, calcium (Ca^2+^) waves, lysosome disruption, mitochondrial damage are the main activators for NLRP3 inflammasome activation in the body [11]. Activation of NLRP3 inflammasome has been documented in the pathogenesis of atherosclerosis, and elevated NLRP3 inflammasome levels were recorded in atherosclerotic patients [12][13][14][15]. However, little is known about the molecular mechanism underlying how NLRP3 inflammasome impacts atherosclerosis.

Recently, emerging studies report that pyroptosis is associated with both NLRP3 inflammasome and atherosclerosis [7][16][17][18][19]. Some studies demonstrate that pyroptosis is a caspase-dependent pro-inflammatory form of programmed cell death, which is characterized by activation of NLRP3 pathways, pore formation of cell membrane, and maturation and release of pro-inflammatory mediators such as interleukin (IL)-1β, IL-18, and pore-forming protein gasdermin D (GSDMD) [20][21][22]. Therefore, this study explores the mediator's role of pyroptosis between NLRP3 inflammasome and atherosclerosis, which may shed light on the molecular mechanism and therapeutic potential in the origination and progression of atherosclerosis.

## NLRP3 INFLAMMASOME

### Components and assembly of NLRP3 inflammasome

**Figure 1:**
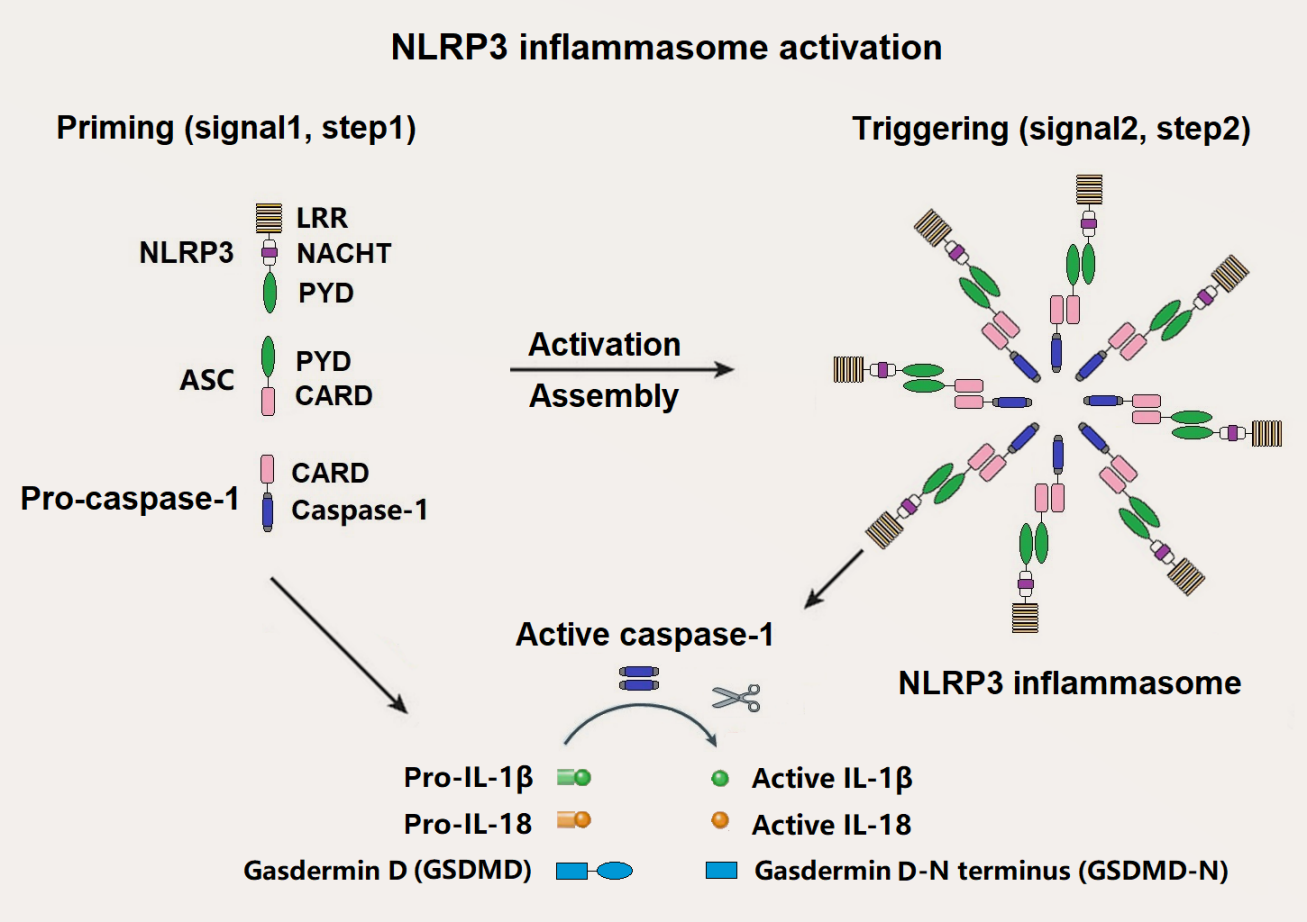
FIGURE 1. Components and assembly of NLRP3 inflammasome. A two-step signal model is proposed for NLRP3 inflammasome. The priming signal (Signal 1, Step 1) induces transcriptional upregulation of pro-IL-1β, pro-IL-18, and NLRP3, which is mediated by PAMPs/DAMPs and induced by post-translational modifications (PTMs) such as de-ubiquitination and phosphorylation. The activation signal (Signal 2, Step 2) is triggered by promoting the oligomerization of inactive NLRP3, ASC, and pro-caspase-1 when the body confronts several intracellular events including K+ efflux, Ca2+ influx, mitochondrial dysfunction such as mitochondrial reactive oxygen species (mtROS) and mitochondrial DNA (mtDNA), lysosomal destabilization, and endoplasmic reticulum stress. Active caspase-1 converts pro-IL-1β, pro-IL-18, gasdermin D (GSDMD) to active IL-1β, IL-18, and GSDMD-N terminus (GSDMD-N), respectively

NLRP3 inflammasome is one of the best known inflammasomes, which is consisted of NLRP3 sensor, ASC adaptor, and caspase-1 effector [10]. NLRP3 consists of three domains: An N-terminal effector pyrin domain (PYD), a central nucleotide-binding and oligomerization (NACHT) domain and a C-terminal leucine-rich repeats (LRRs) domain. ASC is made up of an N-terminal PYD and a C-terminal caspase recruitment domain (CARD). Pro-caspase-1 is composed of a CARD and caspase domains (**Figure 1**). Activated NLRP3 recruits ASC and pro-caspase-1 through a homotypic interaction of PYD-PYD and CARD-CARD, respectively.

### Activation of NLRP3 inflammasome

**Figure 2:**
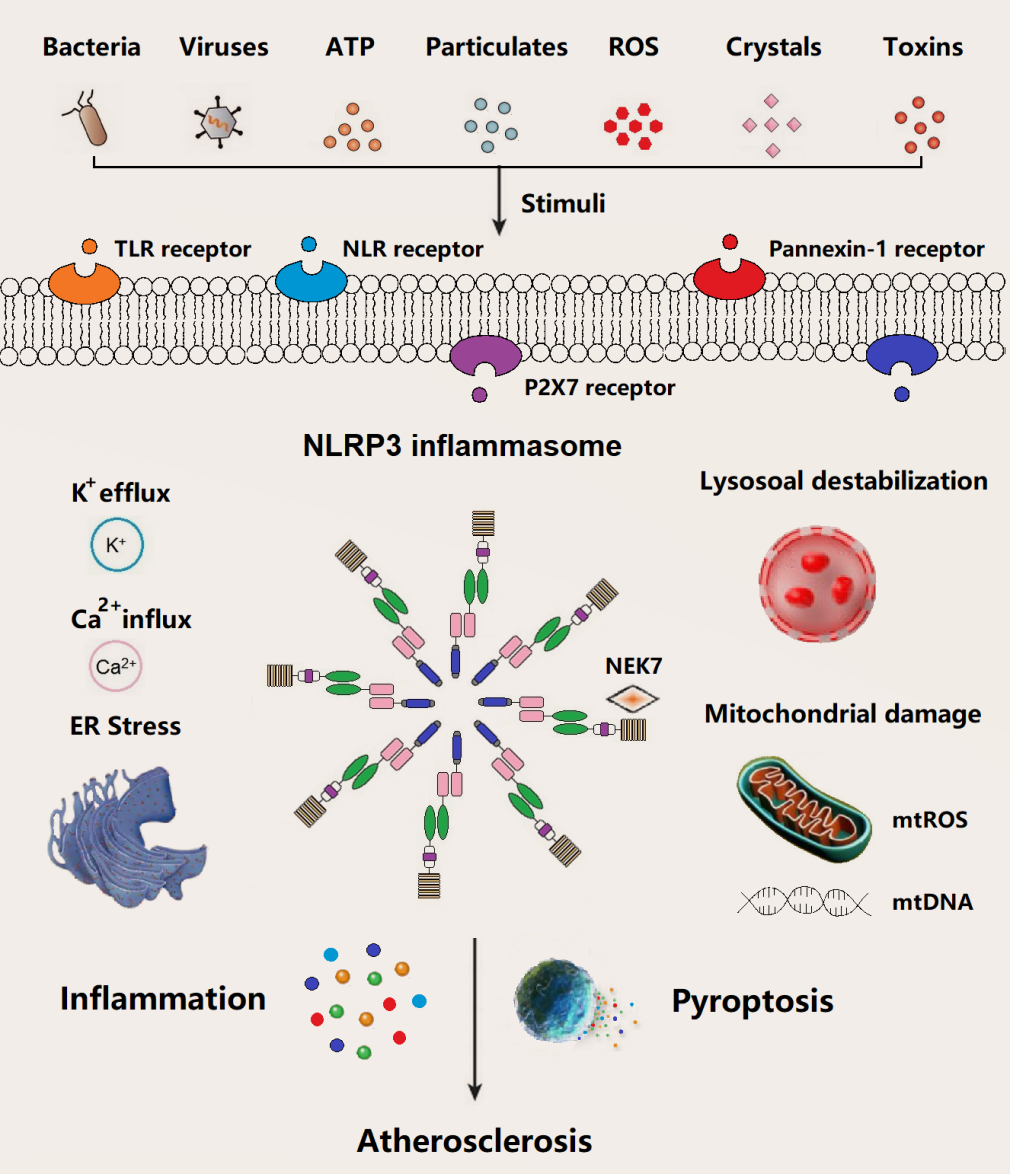
FIGURE 2: Schematic models of haem piracy from haemoglobin by the different Gram positive pathogens described herein. **(A)** In *C. diphtheriae*, two groups of surface exposed proteins bind Hb and Hb:Hp. HtaA is able to bind Hb and the Hb: Hp complex while HtaB is responsible for binding free haem, meanwhile ChtA/ChtC proteins bind directly only to the Hb: Hp complex. Both groups of proteins transfer haem to HmuT, the lipoprotein component of the ABC transporter for import through the lipid bilayer. **(B)** In *S. aureus*, surface exposed proteins IsdB or HarA/IsdH bind haemoglobin at the cell surface and strip it's haem. The haem can them be passed to any of the proteins IsA, IsdC and IsdE and unidirectional transfer from IsaA – IsdC – IsdE leads to transport to the cytoplasm through the IsdDEF ABC transporter for transport into the cytoplasm. **(C)** At the surface of *S. pyogenes*, Shp extracts haem from Hb and Shr is able to obtain haem from both Hb and the Hb:Hp complex. These proteins transfer haem to lipoprotein SiaA for transport into the cell via SiaABC.

In general, the basal expression level of NLRP3 is not enough to activate NLRP3 inflammasome until adequate ASC and pro-caspase-1 are readily in the activated state. NLRP3 inflammasome starts the assembly process in response to infection or sterile inflammation induced by a variety of stimuli such as bacteria, virus, adenosine triphosphate (ATP), particulates, reactive oxygen species (ROS), crystals, and pore-forming toxins (**Figure 2**). Extracellular ATP, particulate matter, crystals, and pore-forming toxins are common up-regulators of NLRP3 inflammasome activation [23]. Above events in relation to NLRP3 inflammasome activation have been documented, mainly including potassium (K^+^) eflux, calcium (Ca^2+^) influx, lysosome destabilization and rupture, mtROS and mtDNA damage, and endoplasmic reticulum (ER) stress. Several intracellular pathways, *i.e.*, nuclear factor κB (NF-κB), mitogen-activated protein kinase (MAPK), and c-Jun N-terminal kinases (JNK) signaling involved in the assembly process of NLRP3 inflammasome, were activated by pathogen-associated molecular patterns (PAMPs) and damage-associated molecular patterns (DAMPs).

## NLRP3 INFLAMMASOME IN ATHEROSCLEROSIS

The main characteristics of atherosclerosis are lipid accumulation, inflammatory cell infiltration, endothelial dysfunction, as well as proliferation of collagen and smooth muscle cells in endarterium [24]. Although dyslipidemia is the leading cause for atherosclerosis, inflammation is also a primary driver and modulator in the occurrence and progression of atherosclerosis. As the best well-known and dominant inflammasomes, NLRP3 inflammasome is involved in the formation and development of inflammation and its related diseases [10]. Several studies have been sprung up in exploring NLRP3 inflammasome in relation to atherosclerosis [25][26][27][28][29][30][31]. However, the results of NLRP3 inflammasome on atherosclerosis are inconsistent and its molecular mechanism is still uncertain.

IL-1β is one of the well-known inflammatory effector molecules during period of NLRP3 inflammasome activation, and also plays a pro-atherogenic role in the progression of atherosclerosis [32]. Therefore, IL-1β has been considered as a key mediator of NLRP3 inflammasome and atherosclerosis. It upregulates the secretion of adhesion molecules in both endothelial cells (ECs) and vascular smooth muscle cells (VSMCs), and accelerates the aggregation of monocytes or macrophages to vascular wall [33][34]. Furthermore, IL-1β could also promote ECs and VSMCs to assemble into the intima and trigger the release of inflammatory cytokines and chemokines to further aggravate inflammation in macrophages [35]. In addition, L-18 share similar function with IL-1β, which tends to be considered as a proatherogenic cytokine in atherosclerosis [36][37][38]. It is worth noting that IL-1β mediates the activation of NLRP3 inflammasome and the pathogenesis of atherosclerosis.

Deficiency of NLRP3, ASC, and IL-1β in bone marrow significantly decreased atherosclerotic lesion formation [39][40][41]. In other words, the above-mentioned bioactive compounds tremendously contribute to development of atherogenesis [35][42][43]. Utilization of gene silencing and specific inhibitors of NLRP3 also demonstrated that there was an atherogenic role of NLRP3 inflammasome [44][45]. Additionally, a reduction in atherosclerotic lesion was found in both low-density lipoprotein receptor (LDLR)^–/–^ mice and apolipoprotein E (ApoE)^−/−^ mice with deficiency in caspase-1/11 [46][47][48]. However, a few studies reported that there was no significant alteration in plaque size or stability of atherosclerosis among ApoE^−/−^ mice no matter whether deficiency of NLRP3, ASC, caspase-1 or not [49], which is inconsistent with the results of the above-mentioned studies. Dissimilar research background and conditions such as sex, atherogenic diet, and hyperlipidemia level may contribute to these conflicting results. For example, insufficiency NLRP3 alleviated atherogenesis in LDLR^−/−^ female mice [40]. Notedly, NLRP3 inflammasome components have been upregulated in human atherosclerosis [14]. Moreover, NLRP3 protein level in peripheral leukocyte were linked with the severity of coronary atherosclerotic patients with acute coronary syndrome [50]. Taken together, NLRP3 inflammasome plays a key role in the development of atherosclerosis.

The underlying molecular mechanisms of NLRP3 inflammasome assemble in atherogenesis have been documented in recent years, such as K^+^ efflux (reduction of intracellular K^+^), Ca^2+^ influx (increase in intracellular Ca^2+^), cathepsin leakage by lysosomal destabilization, ER stress, and mtROS/mtDNA (**Figure 2**) [51][52][53]. Cholesterol crystals (CCs) are one of the most potential stimuli/activators of NLRP3 inflammasome, which is present in atherosclerotic plaques of all stages. The formation of CCs was induced by incorporating oxidized low-density lipoprotein (Ox-LDL) and CD36 scavenger receptor in macrophages, which can activate NLRP3 inflammasome [13][39][54]. Similar to other particle activators like particulate matter, pore-forming toxins, silica, and asbestos of NLRP3 inflammasome, CCs are mainly engulfed/phagocytosed by macrophages and subsequently accumulated in lysosomes. Inadequate digestion of CCs in lysosome may induce lysosomal membrane damage and subsequent leakage of cathepsins into cytoplasm, ultimately result in NLRP3 inflammasome activation [55][56][57]. Meanwhile, along with other particle substances, e.g., nigericin (a bacterial pore-forming toxin and K^+^ ionophore) and extracellular ATP (decrease of intracellular ATP), CCs activate NLRP3 inflammasome via K^+^ efflux pathway accompanied by purinergic P2X_7_ receptor (**Figure 2**) [58]. Additionally, NLRP3 inflammasome assemble can be triggered by calcium phosphate crystals, uric acid crystals, or fatty acid crystals [59][60][61][62]. Extracellular ATP derived from impaired or dead cells make up the necrotic core of atherosclerotic plaque, which triggers the NLRP3 inflammasome through P2X7/K^+^ efflux pathway. P2X7^–/–^/LDLR^–/–^ mice or P2X7-short interference (si) RNA in ApoE^–/–^ mice demonstrated a decline in atherosclerotic lesions [63][64][65]. Hypoxia, a driving factor of atherosclerosis, arose in atherosclerotic plaques, which can induce and enlarge NLRP3 inflammasome activation in macrophage [66][67][68]. Moreover, mtROS/mtDNA can also trigger NLRP3 inflammasome in atherosclerosis [69][70][71]. For example, decreased 8-oxoguanine glycosylase (a DNA glycosylase of eliminating oxidized DNA) in plaque macrophages led to elevated cytosolic oxidized mtDNA and subsequent NLRP3 inflammasome assembly [70]. Notably, NLRP3 inflammasome activation in atherogenesis occurs in a variety of cells such as macrophages, neutrophils, endothelial cells. Several other stimuli such as bacterial, viruses, particle matter, and ROS have been identified to be involved in the priming and triggering NLRP3 inflammasome in the process of atherosclerosis (**Figure 2**).

## PYROPTOSIS IN ATHEROSCLEROSIS

**Figure 3:**
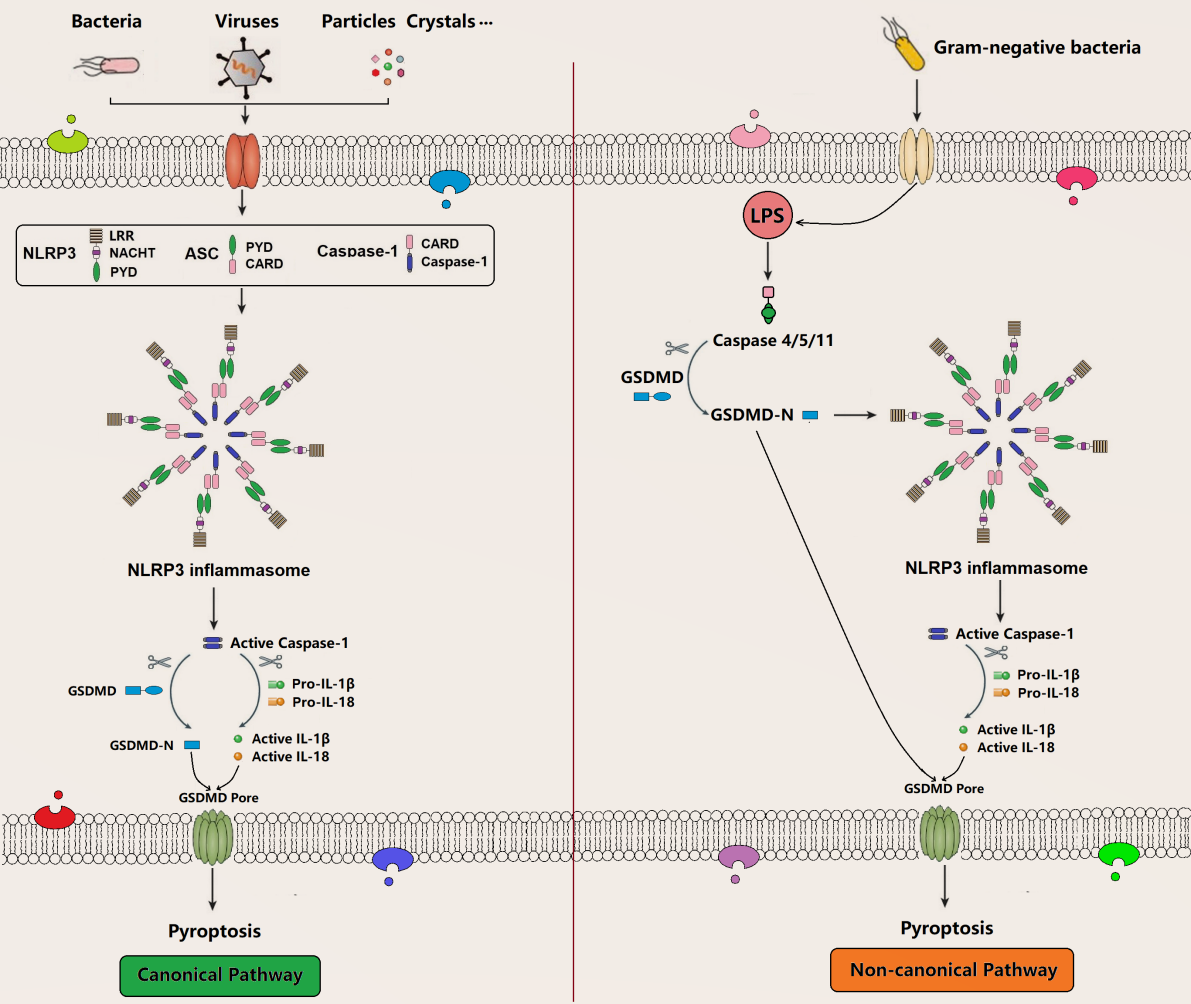
FIGURE 3. Canonical pathway and non-canonical pathway of pyroptosis. The canonical pathway is a caspase1-dependent pyroptosis pathway, and cells can activate inflammatory vesicles to trigger pyroptosis in response to multiple factors that causes caspase-1 activation. It can mature and secret pro-inflammatory cytokines like IL-1β and IL-18 and simultaneously cleave GSDMD and oligomerize GSDMD-N-terminal fragment, which mediates the formation of membrane pores and subsequent pyroptosis. The non-canonical pathway is a caspase 4/5/11-dependent pyroptosis pathway that is activated by the Gram-negative bacterial cell wall fraction LPS, directly triggering pyroptosis through the cleavage of GSDMD. Meanwhile, the GSDMD-N-terminal fragment activates NLRP3 inflammasome to induce pyroptosis. NLRP3: NOD-like receptor protein 3; ASC: adaptor protein termed apoptosis-associated speck-like protein containing an N-terminal pyrin domain (PYD) and a C-terminal caspase recruitment domain (CARD); GSDMD: gasdermin D; LPS: lipopolysaccharide.

Pyroptosis is a recently identified form of programmed cell death, which is characterized by membrane pore formation (bubbling), cell swelling, and cell lysis. Morphologically, it is most likely to be a combination of apoptosis and necrosis. Pyroptosis can be activated by various PAMPs and DAMPs stimuli such as intracellular lipopolysaccharides (LPS), extracellular ATP, cytosolic DNA, bacterial flagella, and particle matters, which is accompanied by inflammasome assembly in terms of caspase-1 (canonical inflammasome pathway) or caspase-4/5/11 (noncanonical inflammasome pathway) that subsequently releases cell contents, secretes proinflammatory cytokines, and induces pyroptosis through the formation of membrane GSDMD pore (**Figure 3**) [72][73][74]. GSDMD is a member of the gasdermin (GSDM) family consisting of a gasdermin-N domain and a unique binding inhibitory domain. Upon activation, it can induce perforation and pyroptosis in mammalian cells. As a substrate of caspase-1, caspase-4, and caspase-5, caspase-11, GSDMD is also an important executor for pyroptosis mediating the pore-forming activity of membrane and the activation signaling of NLRP3 inflammasome [75][76][77][78][79]. There are several molecular pathways in pyroptosis, such as canonical and non-canonical pyroptosis signaling pathway [80].

The pathogenesis of atherosclerosis is characterized by recruitment of monocytes and lymphocytes, dysfunction of ECs, formation of foam cells (FCs), proliferation of smooth muscle cells (SMCs), secretion of proinflammatory cytokines, accumulation and oxidation of LDL, adherence of platelets, and death of abundant cells [7][81]. Previous studies have confirmed that atherosclerosis is mainly attributed to inflammation and the products of pyroptosis such as IL-1β, IL-18, IL-1α, ATP, and GSDMD-N, suggesting crucial role of pyroptosis in the pathogenesis of atherosclerosis [21][82][83]. Pyroptosis is closely in relation to the progress of atherosclerosis [7][16][84]; Additionally, extensive expression of NLRP3 was observed in ECs, macrophages, and SMCs [85]. Therefore, pyroptosis in ECs, macrophage, and SMCs is of a particularly noteworthy concern.

The ECs, locating and lining on the inner surface of vessel walls, are firstly exposure to, and highly susceptible to metabolite-related endogenous danger signals, which plays a critical role in maintaining the fluidity and thromboresistance of blood and regulating the permeability of vascular wall [86]. Previous studies reported that the impairment, loss of intima integrity, or dysfunction of ECs can initiate atherosclerosis [87][88][89]. The dysfunction of ECs is characterized by increased proinflammatory cytokines, elevated reactive oxidative species, chaotic vascular tone, and universal pyroptosis [7]. The activated ECs at the sites of inceptive atherosclerosis up-regulate the expression of P-selectin, intercellular adhesion molecule-1 (ICAM-1) and vascular cell adhesion molecule-1 (VCAM-1), which are critical for attracting inflammatory cells, such as monocytes, for trans-endothelial recruitment [90]. Several cytokines and chemokines, such as IL-1α, ICAM-1, VCAM-1 and E-selectin, in ApoE^-/-^/caspase-1^-/-^ mice were lower than those in ApoE^-/-^ mice [91]. Additionally, pyroptotic ECs contribute to endothelial dysfunction in terms of elevating the permeability of endothelial monolayer and increasing the migration and deposition of lipids, monocytes, and SMCs into the intima accompanied by inflammatory cytokine release, and canonical pathway of NLRP3 is the main mechanism of ECs pyroptosis in atherosclerosis [16].

Foam cells, derived from macrophages, are the main cells engaged in the lesions of atherosclerosis and play a vital role in plaque instability [92]. Monocytes are the most numerous leukocytes in the plaques, which can differentiate into macrophages once they are recruited into arterial intima [21]. The death of macrophages can mediate the development of atherosclerotic lesions, which is manifested by promoting necrotic core formation, increasing plaque vulnerability, and accelerating thrombosis. It is worth pointing out that pyroptosis has been documented to contribute to a substantial proportion of macrophages death in atherosclerotic plaques [93]. Additionally, pyroptosis in atherosclerotic plaques induces inflammation, which leads to the migration of macrophage and SMCs, and the formation of foam cells. Previous studies reported that Ox-LDL and cholesterol crystals could trigger NLRP3 inflammasome and release bioactive IL-1β and IL-18 in macrophages [40]. Moreover, the activation of AIM2 inflammasome and subsequent formation of necrotic cores in macrophage pyroptosis can exacerbate atherosclerosis [94]. Taken together, these studies suggest that NLRP3-dependent pyroptosis in macrophages and foam cells may promote the formation of necrotic core formation and the instability of plaque, and further contribute to the progression of atherosclerosis.

Atherosclerotic plaque rupture is a common and important cause of thrombosis, which could further lead to myocardial infarction [95]. SMCs can produce extracellular matrix to form a fibrous cap through migrating from media layer to intima layer [96]. They are the main cellular components of atherosclerotic lesions, and play a pivotal role in the development and progression of vascular disorders [97]. The stability of atherosclerotic plaque is mainly depended on the thickness of the fibrous cap that is decreased in a state of inflammation [96]. The damage, dysfunction, or death of SMCs and subsequent collapse of extracellular matrix and collagen will thin the fibrous cap, which ultimately results in plaque rupture and complications related to it [98][99]. It has been reported that pyroptosis in SMCs promotes inflammation, which wrecks the fibrous cap [100]. The impairment of fibrous cap aggravates the instability and vulnerability of atherosclerotic plaque and increases the incidence of cardiovascular events [21]. Ox-LDL is a predominant component in atherosclerosis, which can lead to SMCs pyroptosis by activating NLRP3 inflammasome [16]. For example, VX-765, a specific inhibitor of caspase-1, inhibits the pyroptosis of SMCs [101]. Additionally, Ox-LDL induces caspase-1-mediated pyroptosis in SMCs [102]. Taken together, Ox-LDL may accelerate atherosclerotic plaque rupture by triggering SMCs pyroptosis, which may increase the instability of plaques and degrade fibrous cap through the pathway of NLRP3 inflammasome activation.

## RELATION BETWEEN NLRP3 INFLAMMASOME AND PYROPTOSIS

Up-to-date, pyroptosis is a novelty pattern of GSDM-induced pro-inflammatory cell death characterized by the release of a large number of pro-inflammatory factors such as IL-1β and IL-18 in a variety of diseases. Interesting, IL-1β and IL-18 are the typical downstream bioactive products of NLRP3 inflammasome activation based on results of previous studies. A hypothesis was arisen that NLRP3 inflammasome may closely link with pyroptosis. In fact, a plenty of research results confirm the hypothesis in term of coexistence between activation of NLRP3 inflammasome and occurrence of pyroptosis in organism [103][104][105][106][107][108][109]. Many in-depth studies have found the molecular mechanism of NLRP3 inflammasome in relation to pyroptosis. Briefly, external or internal stimuli, such as microorganism, particulates, and ATP, can trigger NLRP3 inflammasome assembly and subsequently mature pro-caspase-1, which ultimately secret IL-1β and IL-18, and induce GSDMD-mediated pyroptosis. Specifically, NLRP3 inflammasome assembly promote pro-caspase-1 activation into active caspase-1, which accelerate pro-inflammatory factor release and GSDMD pore formation in cell membrane, and further induce pyroptosis [103]. NLRP3 inhibitors can alleviate or reverse the activation of NLRP3 inflammasome-mediated pyroptosis [104][105][106][107][108][109]. Blocking NLRP3 and pyroptosis is a new and effective strategy to inhibit inflammation and its related diseases, which may provide a unique perspective to deeply understand the relationship between NLRP3 inflammasome and pyroptosis [110].

## MEDIATION AND THERAPEUTIC TARGETS OF PYROPTOSIS IN NLRP3 INFLAMMASOME AND ATHEROSCLEROSIS

The known signaling pathways of pyroptosis on atherosclerosis are nuclear factors such as NF-κB, AMPK, MAPK, SIRT, as well as miRNA, which may shed light on therapeutic targets for the treatment of atherosclerosis [111]. The vital role of pyroptosis in the pathogenesis of atherosclerosis has generated a few specific inhibitors or agents that target bioactive substances such as NLRP3, caspase-1, caspase-4/5/11, GSDMD, and other candidates in relation to pyroptosis pathway [7][81][84]. Pyroptosis-related atherosclerosis pathway covers the same targeted substances mentioned above, which may suggest that targeting NLRP3 inflammasome may be a therapeutic strategy to treat atherosclerosis. Previous studies have reported that NLRP3 inflammasome inhibitors such as adiponectin, allicin, angiotensin, artemisinin, Bay 11-7082, BOT-4-one, BRC36, CY-09, INF4E, MCC950, OLT1177, and oridonin*, etc* [11][84][112]. The known molecular mechanisms of these inhibitors of NLRP3 inflammasome include the impairment of ATPase activity, prevention of NLRP3 oligomerization, interference of ASC polymerization, obstruction of P2X7 channel, destabilization of the lysosome, or influence ATP/dATP binding in the central NACHT domain [88]. Additionally, caspase-1 inhibitors such as VX-765, Ac-YVAD-cmk, Ac-WEHD-CHO, and Pralnacasan can curb pyroptosis. GSDMD is an imperative pyroptosis executor and the cornerstone of transmembrane channels; the known GSDMD inhibitors, such as necrosulfonamide (NSA), Bay 11-7082 and disulfiram, can also block GSDMD membrane pore formation. Particularly, some pyroptosis associated non-coding RNAs such as miR-223, miR-30–5p, and lncR-MALAT1 have been documented to treat atherosclerosis [96]. General risk factors for atherosclerosis are Ox-LDL, acrolein, and low shear stress, *etc*. Therefore, drugs or agents that target these NLRP3 inflammasome-related substances could be promising or preventing pyroptosis-related diseases such as atherosclerosis.

## CONCLUSION

As a proinflammatory form, pyroptosis plays a crucial role in the pathogenesis and complications of atherosclerosis that mainly targets ECs, macrophages, and SMCs. Pyroptosis in atherosclerotic lesions mainly depends on the NLRP3 inflammasome activation, and the signaling pathways involved provide some potential targets for novel therapeutic interventions in atherosclerosis. There still lacks *in vivo* studies and clinical trials that could provide a solid foundation for developing pyroptosis-inducing drugs. Future studies should concentrate on the molecular mechanisms of NLRP3-mediated pyroptosis in atherosclerosis and other pyroptosis-related chronic diseases.

## AUTHOR CONTRIBUTION

Xiang Zeng: Writing – Original draft, Conceptualization, Supervision, Funding acquisition. Dongling Liu: Writing – review & editing, Conceptualization, Validation, Funding acquisition. Xia Huo: Writing – review & editing, Validation. Yue Wu: Writing – review & editing, Validation. Cuiqing Liu: Writing – review & editing, Validation, Funding acquisition. Qinghua Sun: Writing – review & editing, Validation, Supervision. All authors have read and approved the final manuscript.

## Abbreviatons:

CVDs: – cardiovascular diseases;
NLR: – nucleotide-binding oligomerization domain-like receptor;
NLRP3: – nucleotide-binding oligomerization domain-like receptor family pyrin domain containing 3;
ASC: – apoptosis-associated speck-like protein containing a caspase recruitment domain;
IL: – interleukin;
GSDMD: – gasdermin D;
PYD: – N-terminal effecto pyrin domain;
NACHT: – central nucleotide-binding and oligomerization;
CARD: – C-terminal caspase recruitment domain;
ATP: – adenosine triphosphate;
ROS: – reactive oxygen species;
ER: – endoplasmatic reticulum;
NF-κB: – nuclear factor κB;
MAPK: – mitogen-activated protein kinase;
JNK: – c-Jun N-terminal kinases;
PAMPs: – pathogen-associated molecular patterns;
DAMPs: – damage-associated molecular patterns;
ECs: – endothelial cells;
VSMCs: – vascular smooth muscle cells;
LDLR: – low-density lipoprotein receptor;
ApoE: – apolipoprotein E;
CCs: – Cholesterol crystals;
Ox-LDL: – oxidized low-density lipoprotein;
LPS: – lipopolysaccharide;
FCs: – foam cells;
SMCs: – smooth muscle cells;
ICAM-1: – intercellular adhesion molecule-1;
VCAM-1: – vascular cell adhesion molecule-1;
NSA: – necrosulfonamide.
